# Silica-Calcium-Alginate Hydrogels for the Co-Immobilization of Glucose Oxidase and Catalase to Reduce the Glucose in Grape Must

**DOI:** 10.3390/gels9040320

**Published:** 2023-04-10

**Authors:** David del-Bosque, Josefina Vila-Crespo, Violeta Ruipérez, Encarnación Fernández-Fernández, José Manuel Rodríguez-Nogales

**Affiliations:** 1 Área de Tecnología de los Alimentos, Universidad de Valladolid, Escuela Técnica Superior de Ingenierías Agrarias, 34004 Palencia, Spain; 2 Área de Microbiología, Universidad de Valladolid, Escuela Técnica Superior de Ingenierías Agrarias, 34004 Palencia, Spain

**Keywords:** encapsulation, siliceous material, hybrid capsule, sol-gel network, organic-inorganic gel

## Abstract

Higher temperatures due to climate change are causing greater sugar production in grapes and more alcoholic wines. The use of glucose oxidase (GOX) and catalase (CAT) in grape must is a biotechnological green strategy to produce reduced-alcohol wines. GOX and CAT were effectively co-immobilized by sol-gel entrapment in silica-calcium-alginate hydrogel capsules. The optimal co-immobilization conditions were achieved at a concentration of the colloidal silica, sodium silicate and sodium alginate of 7.38%, 0.49% and 1.51%, respectively, at pH 6.57. The formation of a porous silica-calcium-alginate structure was confirmed by environmental scanning electron microscopy and the elemental analysis of the hydrogel by X-ray spectroscopy. The immobilized GOX showed a Michaelis–Menten kinetic, while the immobilized CAT fits better to an allosteric model. Immobilization also conferred superior GOX activity at low pH and temperature. The capsules showed a good operational stability, as they could be reused for at least 8 cycles. A substantial reduction of 26.3 g/L of glucose was achieved with encapsulated enzymes, which corresponds to a decrease in potential alcoholic strength of must of about 1.5% vol. These results show that co-immobilized GOX and CAT in silica-calcium-alginate hydrogels is a promising strategy to produce reduced-alcohol wines.

## 1. Introduction

Global warming threatens to cause multiple complications due to the foreseeable increase in average temperatures in wine-growing areas and extreme meteorological (torrential rains, hail, drought and frost) and biological (pests and diseases linked to fungi and/or bacteria) phenomena. These problems are already causing, with oscillations year after year, great uncertainty in the management of vineyards due to alterations in the vine and grape cycle that have repercussions on the normal and appropriate production of quality wines [1,2]. One of the most important consequences of higher temperatures is the increase in the concentration of sugars in the grapes, generating an excessive imbalance between technological and phenolic ripening and a higher ethanol content in the resulting wine, which affects its organoleptic characteristics and the winemaking process [3,4].

The phenology of the grapevine is driven by temperature. At higher average temperatures, a significant advance in the vegetative and reproductive cycles of the vine is triggered, anticipating the dates of budbreak, flowering and veraison. Thus, grape ripening (i) starts earlier, (ii) takes place at higher temperatures and (iii) the ripening season is lengthened to reach phenolic ripening. This leads to a higher sugar content and lower organic acid content in the grapes [5]. As it is well known, when must are fermented into wine, yeasts consume the sugars of the grape must and convert them into ethanol and CO_2_. The amount of ethanol produced during fermentation is determined by the amount of sugar present in the must. High sugar and the resulting ethanol concentrations can lead to a number of microbiological, technological, sensory and financial problems [6]. For example, increased sugar concentrations may cause growth inhibition or lysis in microorganisms. This may result in sluggish and stuck alcoholic fermentations or may produce osmotic stress in microorganisms and affect wine quality. Wines with high alcohol content may significantly reduce competitiveness in markets where taxes and/or duties are directly linked to the alcoholic content [3].

This situation requires winegrowers to have technological alternatives according to the peculiarities of their crops and regions. Thus, on the one hand, adaptations in viticulture such as (i) modifications in plant material (grapevine varieties, clones and rootstocks), (ii) vineyard management techniques (grapevine architecture, canopy management, harvest dates, vineyard floor management, timing of harvest, irrigation) and (iii) site selection (altitude, aspect, soil water holding capacity) are being tested to reduce the concentration of sugars in grapes [5]. On the other hand, multiple strategies in the winery during (i) pre-fermentation, (ii) fermentation and (iii) post-fermentation stages are being investigated to achieve a reduction of sugar in must or ethanol in wine with the least possible impact on its organoleptic quality [7,8,9,10,11].

One of these strategies consists of a pre-fermentative treatment of the must with glucose oxidase (GOX) to reduce the concentration of glucose before the alcoholic fermentation, yielding wines with a lower alcoholic strength. At the same time, GOX has the potential to help balance the acidity of must with high pH due to gluconic acid production [12]. Other pre-fermentative strategies are used to reduce the concentration of sugars in the must, such as (i) dilution of the must (illegal in accordance with the wine regulations in certain countries) with reported alteration of mouthfeel and aroma attributes and (ii) membrane filtration techniques (nanofiltration or reverse osmosis) with higher costs and technological adaptation and reported retention of aroma precursors [4,7,8,9].

GOX is a flavoprotein that catalyzes the oxidation of glucose to gluconic acid and hydrogen peroxide (H_2_O_2_) in the presence of oxygen (O_2_). It is common to use GOX in combination with catalase (CAT) to degrade H_2_O_2_ into O_2_ and H_2_O [13]. The generation of O_2_ also allows GOX to use it in a new glucose oxidation cycle. The low pH of the must (between 3.0 and 4.0) limits the glucose conversion by GOX. The relative enzyme activity of GOX from *Aspergillus niger* at pH 5.3 is 100%; however, it is drastically reduced by 50% at pH 3.3 [14,15]. Moreover, the low temperatures of the must in the winery (between 10–25 °C) reduce the activity of this enzyme as its optimum temperature is 40–60 °C [16]. Because of these circumstances, the development of alternatives to enhance the GOX activity under these unfavorable winemaking conditions is necessary. 

One strategy to explore is the enzyme immobilization due to several interesting advantages over the use of free enzymes, such as (i) greater robustness and adaptability under process conditions, (ii) higher efficiency due to the reuse of the biocatalyst, (iii) the possibility of implementing continuous processes in the long term, (iv) better control of the catalysis by an easier separation of the enzyme from the must (avoiding the use of bentonite, which reduces wine quality), (v) faster reaction rates and (vi) improved enzyme stabilization [17].

Enzyme immobilization consists of the physical confinement of the enzyme in a region of space while preserving its enzyme activity. Several techniques have been developed for enzyme immobilization [18]. Entrapment is one of the most widely used due to its simplicity, low cost and mild working conditions. The materials used in enzyme immobilization by entrapment must maintain the enzyme activity, avoid its release and allow the transport of substrates and metabolites. Natural materials (such as alginate, carrageenan, chitosan, agarose, pectin, gelatin and chitin) and synthetic ones have been widely used for the immobilization of numerous enzymes, including GOX and CAT [17,19,20,21,22,23,24,25,26]. Alginate is one of the most widely used polymers due to its lower cost, favorable handling characteristics, biocompatibility, flexibility and elasticity. Alginate is an ionic polymer consisting of 1,4-linked β-D-mannuronic acid and α-L-guluronic acid residues in different sequences. Divalent cations, mainly calcium, put in contact with alginate solutions causing crosslinking of alginate molecules forming hydrogels that can entrap enzymes. However, alginate gels are sensitive to chelating molecules commonly present in food, such as citrates, phosphates and lactates and other antigelling cations, such as sodium and magnesium, reducing their chemical stability [27]. In addition, the low mechanical robustness of calcium-alginate gels is a drawback to be considered in their implementation in the pre-fermentation stage [26]. 

Several strategies have been explored to overcome the disadvantages of using only alginate gels and to improve their mechanical and chemical stability, increase their biocompatibility and control enzyme retention [28]. One of the most outstanding strategies is the integration of other inorganic polymers, such as silica, into alginate structure gel [29]. For silica formation, an aqueous route of the sol-gel process with sodium silicate and colloidal silica has been proposed [30]. This process allows the formation of silica hydrogels at neutral pH and room temperature without the generation of alcohol as a by-product and finally forming with alginate an interpenetrating polymer network (IPN) [31]. The enhancement of the mechanical and chemical stability of the alginate gels with siliceous derivatives has been reported by our research team in oenological conditions. We have generated a hybrid composite that combines the advantages of both the organic alginate polymer and the inorganic silica component (stiffness, chemical resistance and thermal stability) [32,33].

To our knowledge, there are no studies focusing on the design and application of co-immobilized GOX and CAT to reduce glucose concentration in grape must, other than one applied on synthetic must with encapsulated GOX in alginate hollow beads [23]. Our paper describes the development of a new process for the co-immobilization of GOX and CAT in silica-calcium-alginate capsules with the capacity to deplete the glucose from grape must and produce reduced-alcohol wines. The challenge was to develop a matrix that ensured the adequate working conditions for both enzymes. For that, a response surface methodology was employed to optimize the conditions of GOX and CAT co-immobilization. The structural analysis and the elemental chemical composition of the enzyme-containing gels were carried out by environmental scanning electron microscopy and X-ray spectroscopy. The kinetic behavior of the co-immobilized and the free enzymes was studied and compared. pH and temperature profiles under oenological conditions of both types of enzymes were also analyzed. Finally, the operational stability of the encapsulated GOX and CAT and the potential of the immobilized preparation to reduce glucose in must were also evaluated. 

## 2. Results and Discussion

### 2.1. Optimization of the Co-Immobilization of GOX and CAT

In general terms, there are two strategies for the formation of hybrid gels of silica-alginate [34]. The first strategy is the formation of hybrid capsules using a coacervation process. The second one is the coating of the calcium alginate capsules with silica using a layer-by-layer process. In our study, silica nanoparticles and sodium silicate were mixed with sodium alginate, and then this mixture was dripped into a CaCl_2_ solution to obtain a hybrid interpenetrating polymer network of silica-calcium-alginate. We have reported that the hybrid gel prepared with this proposed coacervation process was stronger than the hybrid gel with a silica coating [35]. Therefore, we have decided to utilize the coacervation process for the co-immobilization of GOX and CAT since the use of high-strength gels is a prerequisite for an industrial application of the immobilized enzymes.

Optimizing the immobilization conditions of an enzyme is critical since it is not possible to generalize its immobilization requirements [36]. These requirements depend on the selected immobilization method and matrix, as well as on the enzyme structure and the type of enzyme application. This process is more difficult for a co-immobilization of multiple enzymes since the optimal immobilization conditions for each type of enzyme may be different and even opposites. For this purpose, a reduced Draper-y-Lin composite design was chosen to optimize the variables (concentration of colloidal silica, sodium silicate and sodium alginate, and immobilization pH) that maximize the activity of GOX and CAT in the silica-calcium-alginate hydrogel. This fractional design comprises seven central points for the estimation of the experimental error, and it allows the calculation of the main terms of each variable, the four quadratic terms and the six interaction terms of the quadratic model (Equation (1)) [37]. Table 1 shows the 23 types of enzyme-containing capsules studied and the response variables GC/cap (g of glucose consumed per g of capsules) and HP/cap (g of H_2_O_2_ degraded per g of capsules) determined in each type of capsule. 

**Figure 1:**
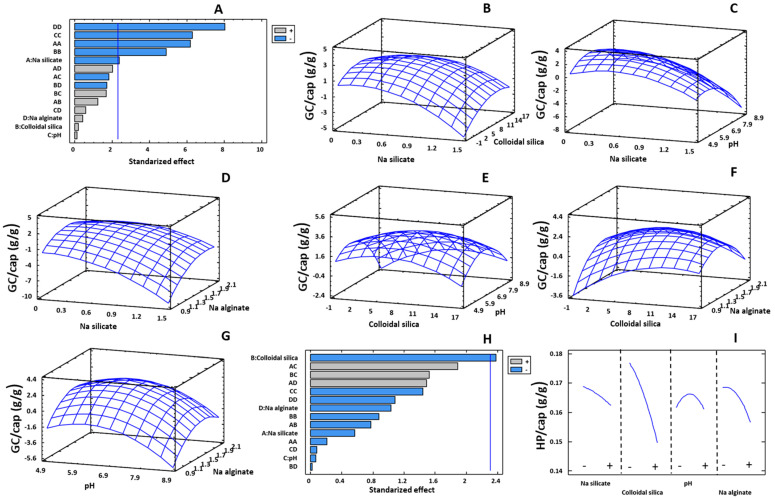
Pareto chart of standardized effect for GC/cap (**A**). Response surface plots (**B**–**G**) for GC/cap as a function of the different variable factors keeping the rest of the variables at their mean level. Pareto chart of standardized effect for HP/cap (**H**). Main effects plot (**I**) for HP/cap (−: low; +: high).

The Pareto chart to GC/cap displaying the standardized effects is illustrated in Figure 1A. The quadratic effects of the concentration of sodium alginate (DD), immobilization pH (CC), sodium silicate (AA) and colloidal silica (BB), as well as the main effect of the concentration of sodium silicate (A) were statistically significant (*p* ≤ 0.05). The analysis of variance determined that the selected quadratic model (Equation (1)) explained 95.9% of the variability of the data. A low coefficient of variation of the assays of the center point of the fractional design (runs 17–23) was obtained (8.35%), indicating a good precision of the experimental data (Table 1). 

Figure 1B–G shows the 3D response surface plots for the GC/cap response, located on the Z-axis, as a function of two variables located on the X- and Y-axes, keeping the rest of the variables at their mean level. The glucose consumption by immobilized GOX was highly influenced by the nature and concentration of the silicon materials, the alginate concentration, as well as the immobilization pH. These parameters could have an effect on the activity of the immobilized GOX due to different phenomena, including modifications (i) in the porosity of the gel, as it affects the transport of substrates and products through the gel pores, (ii) in the enzyme-support interactions and (iii) in the enzyme aggregation and/or unfolding [38,39,40,41,42]. 

The analysis of these plots displayed an optimal value for each variable that maximizes the response in the studied range. Increasing the concentration of colloidal silica, sodium silicate and sodium alginate in the hydrogel enhanced the GC/cap up to the concentrations of 7.38%, 0.49% and 1.51%, respectively. From these values, a decrease in response was observed. Regarding the immobilization pH, the optimum value was obtained at 6.57. At these optimized conditions, a predicted value of 3.39 g of glucose consumed per g of capsules and a predicted immobilization yield of 56.2% were obtained. 

At this optimal hydrogel composition, the porous structure of the gel could be ideal to (i) promote the transport of substrates and products, increasing the efficiency of the GOX catalysis and (ii) decrease the distance between GOX and CAT, increasing the degradation of H_2_O_2_ and reduce the inhibition of GOX by this peroxide [43], as well as to (iii) improve the enzyme trapping. An increase in these variables from their optimal value could cause an excessive rigidity of the immobilization support and a pore diameter too small [44], inducing a crowded effect and reducing the mobility of the enzymes and substrates, and therefore an impaired the enzyme activity [45]. It has been reported that increasing the alginate concentration reduced GOX release, but it may cause substrate and product transport difficulties as the porosity of the gel decreases [46]. It can also be observed that the optimal immobilization pH value (6.57) was close to the optimal pH range for GOX from *A. niger* (4.5–6.5) [16,47].

Regarding CAT, the Pareto chart showed that only the concentration of colloidal silica was statistically significant (*p* ≤ 0.05) (Figure 1H). In the selected working range, the sodium silicate and sodium alginate concentrations and the immobilization pH did not influence the HP/cap response. The analysis of variance of the data showed that R^2^ (84.57%) was lower than the one observed for CG/cap though a similar value for the coefficient of variation of the center trials (9.31%) was achieved, also denoting a low experimental error. Figure 1I shows that an increase in the colloidal silicate concentration reduced the capacity of the immobilized CAT to decompose H_2_O_2_. As described for GOX, this effect could also be attributed to diffusion problems of substrate and/or unfavorable interactions between the enzymes and the immobilization support. Bower et al. [48] reported that the adsorption of enzymes on colloidal silica induced a reduction in their enzyme activity, probably caused (i) by a change in the structure and orientation of the enzymes and (ii) by electrostatic effects. For CAT, using the mathematical model of Equation (1) (see Section 4.2), the maximum response was obtained at a concentration of colloidal silicate and sodium alginate of 3.69% and 0.98%, respectively, and an immobilization pH of 5.34 and without sodium silicate, achieving a predicted value of 0.221 g of H_2_O_2_ degraded per g of capsules and a predicted immobilization yield of 16.1%.

Considering that the goal of this study was to optimize the conditions for the co-immobilization of GOX and CAT to maximize glucose consumption and that the variation of enzyme entrapment conditions resulted in greater changes for GC/cap than for HP/cap, we decided to perform the co-immobilization of both enzymes under the optimal conditions achieved for GOX.

### 2.2. Morphological Observations and Chemical Characterization of the Capsules

**Figure 2:**
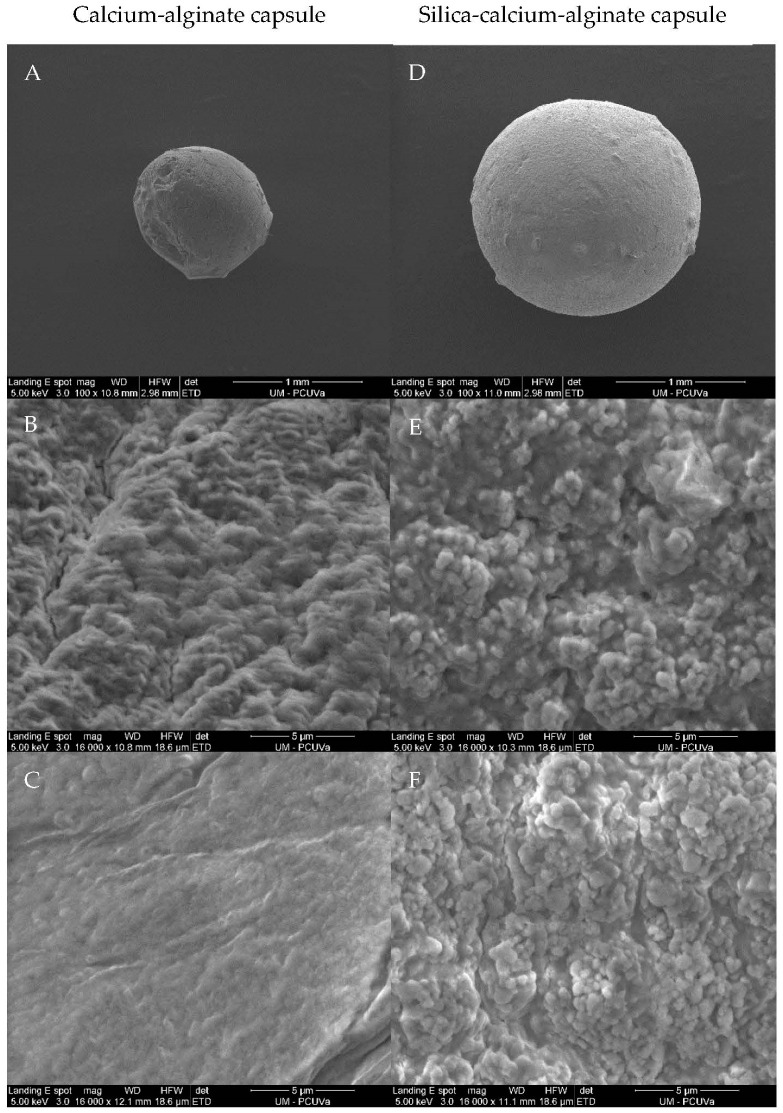
Scanning electron microscopy micrographs of external (**A**,**B**,**D**,**E**) and internal (**C**,**F**) surfaces of calcium-alginate (**A**–**C**) and silica-calcium-alginate (**D**–**F**) capsules.

The morphological observations of calcium-alginate and silica-calcium-alginate capsules are shown in Figure 2. Dry calcium-alginate (A) and silica-calcium-alginate (D) capsules were nearly spherical, with a diameter of ~1.10 and ~1.76 mm, respectively. The presence of sodium silicate and colloidal silica in the composition of the capsule increased its diameter as a result of an enhancement in the viscosity of the sol-gel solution [33]. The external surface of the alginate capsule was relatively rough and porous (B), although its internal surface was smoother and more homogeneous (C). The morphology of the capsule with siliceous material was rougher and more porous (E, F), both on the external and internal surfaces. These results are in accordance with those found by Simó et al. [33] and Jung et al. [44] An increase in the porosity of the immobilization support enhances the mobility of substrate molecules towards the immobilized enzyme [49,50].

**Figure 3:**
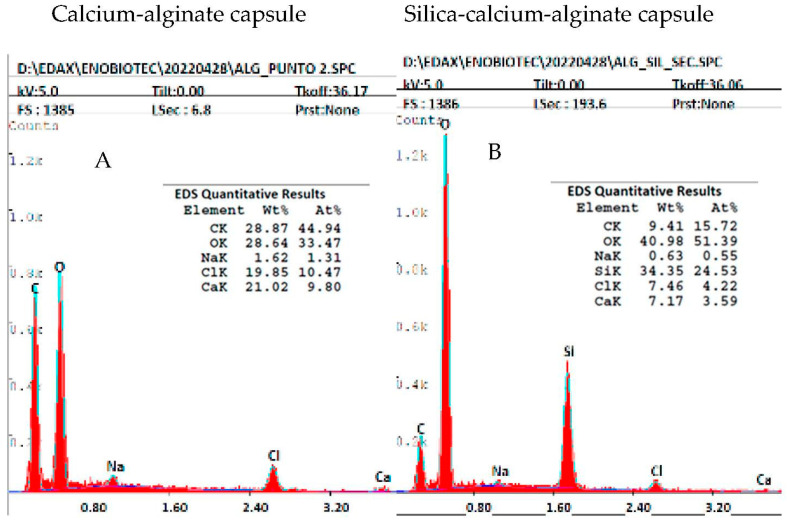
Microstructural characterization of calcium-alginate (**A**) and silica-calcium-alginate (**B**) capsules.

The elemental analysis of calcium-alginate and silica-calcium-alginate capsules was carried out by X-ray spectroscopy. Figure 3A shows that the element peaks of the calcium-alginate capsule included carbon, oxygen, sodium, chloride and calcium. Chloride and calcium elements were from the calcium chloride solution used for the gelation of sodium alginate, and carbon, oxygen, and sodium from the chemical composition of the sodium alginate. The element peaks of the silica-calcium-alginate capsule (Figure 3B) included carbon, oxygen, sodium, silicon, chlorine and calcium. The peak of silicon verified the presence of SiO_2_ in this capsule from colloidal silica and sodium silicate. The highest relative number of atoms was observed for the silicon atom (24.53%), indicating that the siliceous material was the main component of this capsule. The relative number of carbon atoms decreased from 44.94% in the calcium-alginate capsule to 15.72% in the silica-calcium-alginate capsule. On the contrary, the relative number of oxygen atoms increased from 33.47% to 51.39%, respectively. These changes in the relative number of carbon and oxygen atoms are due to the incorporation of colloidal silica and sodium silicate in the hydrogel of calcium alginate.

### 2.3. Kinetic Analysis

**Figure 4:**
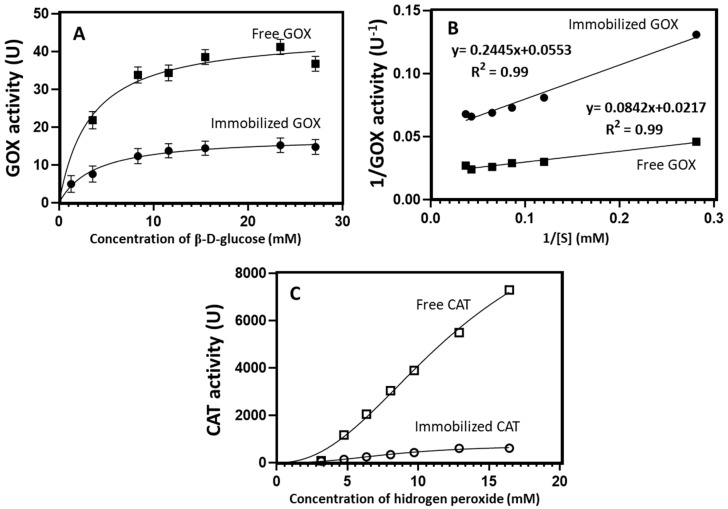
Michaelis–Menten curve (**A**) and Lineweaver–Burk plot (**B**) for the determination of kinetic parameters of free and immobilized GOX. Velocity curve as a function of the substrate (**C**) showing the allosteric sigmoidal behavior of free and immobilized CAT.

To study the effect of co-immobilization on GOX kinetic, the initial reaction rates of free and immobilized GOX were measured at different glucose concentrations (1.0 to 27.0 mM) (Figure 4A). The experimental data of free and immobilized GOX fit Equation (2) very well (R^2^ = 0.99) (Figure 4B), suggesting that both types of enzymes displayed a Michaelis–Menten-like kinetic. The apparent *K_m_* of the immobilized GOX (4.42 mM) was slightly higher than that of the free enzyme (3.88 mM), indicating that the hydrogel of silica-calcium-alginate partly limited the permeation rate of glucose through the porous capsule [30]. Rodriguez-Nogales (2004) [26] suggested that chemical and/or conformational changes in GOX by an association of the enzyme with the capsule materials could also possibly increase the apparent *K_m_*. In contrast, the *V_max_* of immobilized GOX (18.08 U) was smaller than that observed for free GOX (46.08 U), probably because of reduced glucose transport into the capsule. The values observed for *K_m_* and *V_max_* suggest that the immobilization process partially reduced the GOX activity.

The kinetic behavior of free and immobilized CAT was studied by evaluating the initial reaction rates of the enzyme at different H_2_O_2_ concentrations (3 to 16 mM) (Figure 4C). Free and immobilized CAT did not show a Michaelis–Menten kinetic they exhibited a cooperative allosteric kinetic instead, as was described by Sun et al. (2017) [51]. An R^2^ of about 0.99 was obtained by fitting the experimental data to equation 3 of an allosteric kinetic for both free and immobilized CAT. Immobilized CAT presented a lower $K_{half}^{h}$ (8.41 mM) than that observed for the free enzyme (13.08 mM), pointing out that a smaller concentration of H_2_O_2_ was required to obtain a half-maximal enzyme velocity. On the contrary, the Hill slope (*h*) of immobilized CAT was higher (2.60) than that of the free enzyme (2.24). Finally, a decrease in the *V_max_* of CAT caused by the immobilization of the enzyme into the silica-calcium-alginate hydrogel was observed (11,397 and 742 U for free and immobilized CAT, respectively), probably due to the substrate diffusion problems into the capsule. 

### 2.4. Effect of pH and Temperature under Oenological Conditions

To determine the optimal pH values under oenological conditions for the co-immobilized GOX and CAT and compare them with the optimal pH of the free enzymes, the pH of the substrate solutions was changed between 3.0 and 4.0. Free GOX showed higher relative catalytic activity at pH values equal to or higher than 3.8 and decreased significantly in the lower pH range (3.0–3.4) (Table 2), whereas the activity of the immobilized GOX exhibited higher relative activity at pH range between 3.2 and 3.8. In the case of CAT, the relative activity of the free and immobilized enzyme was maximum at pH 4.0, with a moderate decrease at the pH below 4.0 for the free enzyme and a more pronounced decrease for the immobilized one. These results display that silica-calcium-alginate gels shifted the GOX local optimum pH towards a more acidic pH likely because of the reported buffering capacity of the alginate [52]. On the contrary, the immobilized CAT had worse adaptation to the pH changes that the free CAT. It has been described that immobilized enzymes can exhibit different behavior at different pH according to the type of enzyme as well as the nature of the immobilization support [53].

Regarding the effect of temperature on GOX activity (Table 2), the highest relative activity for the free enzyme was observed in the temperature range of 20–25 °C. However, the immobilized GOX had higher relative catabolic activity at the lowest assayed temperature (10 °C), which can prevent a loss of enzymatic activity during a prolonged operational period. Enhanced stability of the entrapped GOX in the calcium-alginate gel has been also reported at low temperatures [54]. The diffusion of O_2_ was probably higher at lower temperatures since the solubility of this gas in an aqueous media increases as temperature decreases [55]. The observed decrease in the immobilized GOX activity as temperature increases may be caused by higher diffusion limitations [56], which are not balanced by the increased enzyme activity at a higher temperature. Based on these results, the immobilized GOX could be effectively applied in musts at low temperatures. From an oenological point of view, these conditions reduce the onset of alcoholic fermentation and, therefore, the CO_2_ production associated with this process, allowing the oxidative activity of GOX.

The activity of the free CAT enzyme was quite similar over the range of temperatures tested. However, a higher activity for immobilized CAT was reached at 20 °C. In this case, the silica-calcium-alginate gel did not improve the CAT activity at low temperatures, unlike that observed for GOX at 10 °C.

### 2.5. Operational Stability of the Immobilized GOX and CAT

**Figure 5:**
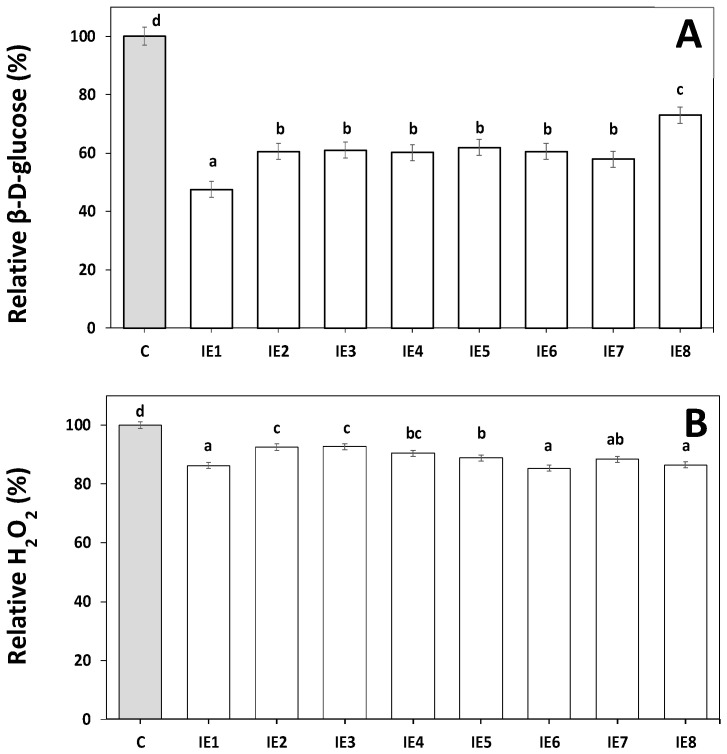
Operational stability of co-immobilized GOX (**A**) and CAT (**B**) capsules at 25 °C in 8 consecutive cycles of 90 min and 2 min, respectively. C: Control without enzymes; IEn: Immobilized enzymes (n = cycle number). Bars with different letters are significantly different at *p* ≤ 0.05.

The success of any industrial enzyme application depends to a large extent on the maintenance of the enzyme activity during the operating time. The use of an immobilized enzyme with high operational stability reduces the cost of its application [57]. The operational stability of GOX and CAT was evaluated by assaying the consumption of glucose or degradation of H_2_O_2_, respectively, in 8 consecutive cycles of reusing the capsules. Although a higher relative activity of the immobilized GOX was obtained at 10 °C, we decided to evaluate its operational stability at the least favorable temperature of 25 °C. Figure 5A shows good operational stability of the immobilized GOX, showing a consumption of 52.4% of the initial glucose concentration in the first cycle. From the second cycle on, the efficiency of the enzymatic process was still high (~40%). This moderate decline in GOX activity could be caused by several reasons, such as (i) a loss of the enzyme during the assay, (ii) a reduction of the GOX activity due to an accumulation of gluconic acid inside the gel matrix lowering the pH of its aqueous phase and/or (iii) an accumulation of H_2_O_2,_ which is a known GOX inhibitor [43,58].

Similar to our results, Ruiz et al. (2018) [23] reported that the encapsulated GOX in alginate hollow beads was reusable for at least eight cycles with a final reaction efficacy of 37%. Co-immobilized GOX and CAT in a hybrid interpenetrating polymer network based on alginate could be reutilized up to four times with a high glucose conversion [43]. Regarding the immobilized CAT (Figure 5B), high operational stability of the immobilized CAT was also observed, allowing its reuse for at least eight cycles and maintaining its initial degradation capacity. This performance could be attributed to the protective effect of the silica-calcium-alginate gel against CAT denaturation and the low reaction time used in each cycle that would reduce the enzyme loss. High operational stability of CAT in alginate gels has been recently reported by Czyzewska andTrusek (2023) [59].

### 2.6. Reduction of Glucose in Must with the Co-Immobilized GOX and CAT 

**Figure 6:**
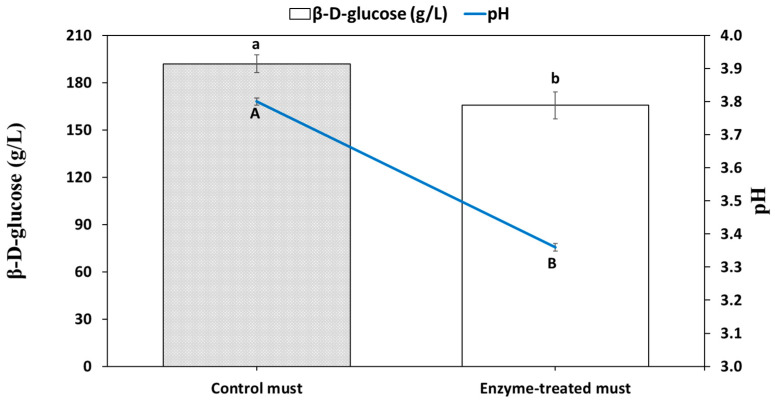
Glucose concentration and pH of the control and the enzyme-treated musts. Bars with different letters are significantly different at *p* ≤ 0.05.

The treatment of the must with the co-immobilized enzymes resulted in glucose consumption of 26.3 ± 0.2 g/L (Figure 6). Together with this glucose depletion, a decrease in the initial pH of the must from 3.8 to 3.4 was observed, caused by the conversion of glucose to gluconic acid [14]. The coefficients of variation of glucose consumption and pH reduction were very small (0.7% and 0.3%, respectively), indicating a low variability among trials. A decrease of ~1.5% vol. (*v*/*v*) in the potential alcoholic strength of the must was achieved, estimating that 1.0% vol. (*v*/*v*) corresponds to a consumption of ~17 g/L of glucose by yeasts during alcoholic fermentation [11]. This reduction is in line with those accepted in the International Organization of Vine and Wine (OIV) using separation techniques, such as partial vacuum evaporation, membrane techniques and distillation [60], which indicates that alcohol reductions over 20% of the initial value are not allowed (e.g., 2% degrees for a wine initially around 10%).

## 3. Conclusions

An effective immobilization support based on an interpenetrated polymer network of silica-sodium-alginate hydrogels was developed for the co-immobilization of GOX and CAT. This hydrogel showed a highly porous structure and enhanced GOX activity at low pH and temperatures. The control of the polymerization conditions was crucial for GOX and less so for CAT. The capsules showed a satisfactory behavior regarding their operational stability, as they could be reused for at least eight cycles. Moreover, the co-immobilization of GOX and CAT in silica-calcium-alginate hydrogels could be a very advantageous strategy to produce reduced-alcohol wines. A significantly decreased concentration of glucose in must was observed, which could reduce the excessive wine alcohol degree with a negative impact on its sensory quality and avoid the risk of stuck or sluggish fermentations. In addition, these immobilized enzymes could be very attractive for the biotechnological industry that demands new productive biocatalysts and new strategies to increase the useful life of enzymes. Further studies are required to test the technical feasibility of the silica-calcium-alginate immobilized GOX and CAT as a green strategy to reduce the alcoholic strength of the wine.

## 4. Materials and Methods

### 4.1. Enzymes and Chemical Reagents

Glucose oxidase (GOX, EC 1.1.3.4, Gluzyme^®^ Mono 10.000 BG from *A. niger*, 10 KU/g) and catalase (CAT, EC 1.11.1.6, Catazyme^®^ 25 L from *A. niger*, 25 KU/mL), were kindly provided by Novozymes (Bagsvaerd, Denmark). LUDOX^®^ HS-40 colloidal silica (420816) and sodium silicate (338443) were purchased from Sigma-Aldrich (St. Louis, MO, USA). Sodium alginate (A3249), β-D-glucose (A1422) and H_2_O_2_ (33%, *w*/*v*) (131,077.1211) were purchased from Panreac Applichem (Darmstadt, Germany). The rest of the chemicals were analytical quality grade and purchased from Panreac, S.A. (Madrid, Spain).

### 4.2. Optimization of the Co-Immobilization of GOX and CAT in Mixed Silica-Calcium-Alginate Capsules

Co-immobilization of GOX and CAT was carried out using the IPN method by entrapment in hybrid silica-calcium-alginate hydrogels. This method is based on the mixture of silicon derivatives with sodium alginate prior to gelation in the presence of Ca^2+^ [61,62]. The co-immobilization of GOX and CAT was optimized using a response surface methodology. The concentration of colloidal silica (0.0–15.1%), sodium silicate (0.3–1.3%) and sodium alginate (1.0–2.1%), as well as the immobilization pH (4.9–8.0) were optimized. A reduced Draper-y-Lin composite design, rotatable, orthogonal and quadratic processed, with 7 central points for the estimation of the experimental error, was chosen to generate 23 experiments.

The commercial GOX (Gluzyme^®^ Mono 10.000 BG) contains a matrix of wheat flour that could interfere with the immobilization process. Therefore, a flour-free enzyme extract was prepared by dissolving 7.5 g of Gluzyme^®^ Mono in 25.0 mL of 0.1 M citrate buffer at pH 6.6 with stirring at 225 rpm (Orbital Shaker SO1, Stuart Scientific, Stone, UK) for 30 min at 30 °C. The resulting solution was centrifuged at 2320× *g* for 15 min (Sorvall ST 8R Centrifuge, Osterode am Harz, Germany) and the supernatant was used as source of GOX (Solution 1). CAT was taken directly from the commercial enzyme preparation (Solution 2). 

On the one hand, colloidal silica and sodium silicate were dissolved together in distilled water at room temperature and the appropriate pH was adjusted with 1 N HCl (Solution 3). On the other hand, sodium alginate was dissolved at 40 °C in distilled water (pH previously adjusted with 1N HCl) (Solution 4). Solutions 3 and 4 were then mixed and kept under stirring at 1200 rpm for 3 min. 0.1 mL of Solution 1 (GOX) and 0.012 mL of Solution 2 (CAT) were added per mL of this homogenous mixture achieving a concentration of 117 U of GOX and 23 kU of CAT per mL of the final mixture. Stirring was maintained at 1200 rpm for 3 min until a complete homogenization. This final mixture was dripped with a 10 mL syringe (BD Plastipak, Toledo, Spain) (at a height of 20 cm) into a 0.2 M CaCl_2_ solution and kept in agitation at 300 rpm for 60 min. After this period, the capsules obtained were kept under refrigeration at 4 °C for 24 h in 0.2 M CaCl_2_. The two dependent response variables were (i) g of glucose consumed per g of capsule (GC/cap) to assess the activity of the immobilized GOX and (ii) g of H_2_O_2_ degraded per g of capsule (HP/cap) to evaluate the activity of the immobilized CAT. 

GC/cap was estimated by incubating 5 mL of β-D-glucose (20 mM) dissolved in sodium acetate buffer (0.1 M, pH 5.1) with 15 capsules (~0.40 g) for 90 min at 25 °C under agitation at 150 rpm. The initial and final β-D-glucose concentrations were measured with an enzyme kit (K-FRGLQR-02/17 Megazyme Bray Co., Wicklow, Ireland). HP/cap was estimated by incubating 10 mL of H_2_O_2_ (0.05 %, *w*/*v*) dissolved in phosphate buffer (0.07 M, pH 7.0) with 15 capsules for 2 min at 25 °C under agitation at 150 rpm. The initial and final H_2_O_2_ concentrations were measured by absorption spectroscopy at 240 nm [63]. 

One unit of GOX or CAT activity was expressed as the amount of free or immobilized enzyme required to transform 1 mM of glucose or H_2_O_2_, respectively, per min using the aforementioned assay conditions. The immobilization yield (in %) was calculated as ratio between the units of GOX or CAT activity immobilized to the units of GOX or CAT used initially for the immobilization.

With the data of each assay for each dependent response variable, the optimized conditions were established using Statgraphics Centurion (v.19, Statgraphics Technologies, Inc. The Plains, VA, USA) fixing a second-order model for the independent variables with a significance level (α) of 0.05 and 15 coefficients as shown in Equation (1) [64].

(1) $$y=\beta_{o}+\sum_{i=1}^{k}\beta_{i}X_{i}+\sum_{i=1}^{k}\beta_{ii}X_{i}^{2}+\sum_{i}^{i<j}\;\sum_{j}\beta_{ij}X_{i}X_{j}+\varepsilon$$

where, y is a dependent response variable, $X_{i}$ and $X_{j}$ are the 4 independent factors, $\beta_{o}$, $\beta_{i}$, $\beta_{ii}$ and $\beta_{ij}$ are the regression coefficients, and ε is the error. The outcome of the ANOVA can be visualized in a Pareto Plot, where the absolute value of the standardized estimated effect of each factor investigated on a dependent response variable is plotted.

### 4.3. Structural and Compositional Analysis of the Capsules

The structure of the capsules was analyzed by environmental scanning electron microscopy in a Quanta 200FEG ESEM (Hillsboro, OR, USA). For this purpose, silica-calcium-alginate and calcium-alginate capsules with co-immobilized GOX and CAT were left to dry before analysis. These samples were examined in low vacuum (LV) mode with a large field electron detector. Additionally, the composition of the capsules was evaluated by environmental scanning electron microscopy coupled with energy-dispersive X-ray spectroscopy.

### 4.4. Determination of the Kinetic Parameters of the Immobilized and Free GOX and CAT

The kinetic parameters for both the immobilized and the free GOX were calculated using 5 mL of β-D-glucose at different concentrations in the range of 1.0 to 27.0 mM dissolved in sodium acetate buffer (0.1 M, pH 5.1). Each assay was performed at 25 °C for 5 min. The kinetic parameters for the immobilized and the free CAT were calculated using 10 mL of H_2_O_2_ in the range of 3 to 16 mM dissolved in phosphate buffer (0.07 M, pH 7.0). Each assay was performed at 25 °C for 30 s. All assays were carried out under agitation at 150 rpm, in triplicate, and using 15 capsules for co-immobilized GOX and CAT, and the concentration of free enzymes corresponding to 15 capsules. $K_{m}$ and $V_{max}$ values for GOX were calculated using a plot according to the following Lineweaver–Burk Equation (2) [65]:

(2) $$\frac{1}{V_{O}}=\frac{Km}{V_{max}}\cdot \left[S\right]+\frac{1}{V_{max}}$$

where $V_{o}$ is the initial velocity, *K_m_* is the Michaelis–Menten constant, $V_{max}$ is the maximum velocity and $\left[S\right]$ is the different substrate concentrations. 

The kinetic data for CAT showed a positive cooperative sigmoidal allosteric behavior of the enzyme. $K_{m}$ and $V_{max}$ values for CAT were calculated using a plot according to the following Equation (3):

(3) $$V_{o}=V_{max}\;\frac{{\left[S\right]}^{h}}{\left(K_{half}^{h}+{\left[S\right]}^{h}\right)}$$

where $K_{half}^{h}$ is the concentration of substrate that produces a half-maximal enzyme velocity, and *h* is the hill slope. When *h* = 1.0, this equation is identical to the standard Michaelis–Menten equation. When it is bigger than 1.0, the curve is sigmoidal due to positive cooperativity. The calculations were performed with GRAPHPAD Prism 9 (v. 9.3.0, San Diego, CA, USA).

### 4.5. Effects of pH and Temperature in the Immobilized and Free GOX and CAT

The effect of pH on both free and co-immobilized GOX and CAT was assayed using β-D-glucose (20 mM) solution dissolved in sodium acetate buffer (0.1 M) or H_2_O_2_ (0.05%, *w*/*v*) dissolved in phosphate buffer (0.07 M), respectively, at six different pH values in the oenological range of 3.0 to 4.0. The effect of temperature was studied using four different temperature values in the range of 10 to 25 °C found in a winery. The other experimental conditions employed for the determination of GC/cap and HP/cap were used. For the free GOX and CAT, the concentration of free enzyme corresponding to 15 capsules was assayed. All pH and temperature assays were carried out in triplicate.

### 4.6. Operational Stability of the Capsules

The operational stability of the co-immobilized GOX and CAT was determined by quantifying the glucose consumed in 5 mL of β-D-glucose (20 mM) in sodium acetate buffer (0.1 M, pH 5.1) or 10 mL of H_2_O_2_ (0.05 %, *w*/*v*) in phosphate buffer (0.07 M, pH 7.0) in 8 consecutive cycles of 90 min or 2 min each, respectively, with repeated use of the capsules. Fifteen capsules were used and washed after each cycle with 30 mL of the corresponding buffer for 30 s before starting the next cycle. Values were expressed as relative percentage of β-D-glucose for GOX and relative percentage of H_2_O_2_ for CAT. All assays were performed at 25 °C, shaking at 150 rpm, in triplicate.

### 4.7. Treatment of Must with the Co-Immobilized GOX and CAT

Three 50-mL Falcon tubes with 5 mL of grape must (191.9 g/L of glucose) at pH 3.8, centrifuged at 2320× *g* for 5 min, were treated with 15 capsules and maintained in an orbital shaking at 150 rpm for 48 h at 15 °C. A control must without capsules was maintained at the same conditions. After the treatment, the concentration of glucose and pH were measured in musts in triplicated. 

### 4.8. Statistical Analysis

The analysis of the experimental design and variance of the experimental data were performed at a *p* ≤ 0.05 using the Stratigraphic 19 Centurion statistical package (v.19, Statgraphics Technologies, Inc., The Plains, VA, USA).

## Figures and Tables

**Table 1:** Reduced Draper-y-Lin composite design with the 23 types of enzyme-containing capsules and the two response variables.

	Factors				Responses ^1^	
Assay	Sodium Silicate (%)	Colloidal Silica (%)	Immobilization pH	Sodium Alginate (%)	GC/Cap (g/g)	HP/Cap (g/g)
1	0.6	7.5	8.01	1.5	1.74 ± 0.15	0.15 ± 0.00
2	0.6	7.5	4.99	1.5	1.95 ± 0.21	0.16 ± 0.01
3	0.3	3.1	7.40	1.2	1.98 ± 0.17	0.17 ± 0.00
4	0.6	15.1	6.50	1.5	2.19 ± 0.19	0.14 ± 0.01
5	1.0	3.1	5.60	1.9	1.89 ± 0.27	0.17 ± 0.00
6	0.6	7.5	6.50	2.1	1.56 ± 0.19	0.15 ± 0.00
7	0.6	0.0	6.50	1.5	2.11 ± 0.21	0.18 ± 0.00
8	0.0	7.5	6.50	1.5	2.38 ± 0.21	0.17 ± 0.00
9	1.0	3.1	7.40	1.9	1.38 ± 0.26	0.18 ± 0.01
10	0.3	12.0	7.40	1.9	1.54 ± 0.31	0.13 ± 0.01
11	1.0	12.0	5.60	1.2	1.33 ± 0.24	0.11 ± 0.01
12	0.3	3.1	5.60	1.2	1.97 ± 0.33	0.20 ± 0.01
13	0.6	7.5	6.50	1.0	1.38 ± 0.22	0.17 ± 0.01
14	1.3	7.5	6.50	1.5	1.36 ± 0.21	0.16 ± 0.01
15	0.3	12.0	5.60	1.9	0.55 ± 0.25	0.13 ± 0.01
16	1.0	12.0	7.40	1.2	1.29 ± 0.21	0.14 ± 0.01
17	0.6	7.5	6.50	1.5	3.66 ± 0.35	0.18 ± 0.00
18	0.6	7.5	6.50	1.5	3.07 ± 0.26	0.17 ± 0.00
19	0.6	7.5	6.50	1.5	3.23 ± 0.29	0.18 ± 0.00
20	0.6	7.5	6.50	1.5	3.08 ± 0.26	0.18 ± 0.00
21	0.6	7.5	6.50	1.5	3.31 ± 0.15	0.15 ± 0.09
22	0.6	7.5	6.50	1.5	3.78 ± 0.16	0.16 ± 0.10
23	0.6	7.5	6.50	1.5	3.53 ± 0.16	0.15 ± 0.10

^1^ GC/cap are g of glucose consumed per g of the capsule (incubation time of 90 min at 25 °C and pH 5.1) to assess the activity of the immobilized GOX, and HP/cap are g of H_2_O_2_ degraded per g of the capsule (incubation time of 2 min at 25 °C and pH 7.0) to evaluate the activity of the immobilized CAT. Each value represents the mean ± its 95% confidence interval.

**Table 2:** Effect of pH and temperature (T) on free and immobilized GOX and CAT activity.

		Relative GOX Activity (%)		Relative CAT Activity (%)	
		Free GOX	Immobilized GOX	Free CAT	Immobilized CAT
pH	3.0	69.47 ± 3.28 ^a^	71.72 ± 7.87 ^a^	86.23 ± 0.88 ^a^	38.41 ± 9.22 ^a^
	3.2	69.79 ± 3.41 ^a^	89.28 ± 8.16 ^b^	87.38 ± 0.89 ^ab^	56.81 ± 9.40 ^ab^
	3.4	72.14 ± 3.42 ^a^	98.67 ± 8.21 ^b^	87.06 ± 0.90 ^a^	60.71 ± 9.60 ^b^
	3.6	87.88 ± 3.52 ^b^	100.00 ± 8.40 ^b^	87.37 ± 0.93 ^ab^	73.19 ± 10.04 ^bc^
	3.8	92.82 ± 3.58 ^bc^	92.65 ± 8.59 ^b^	89.08 ± 0.97 ^b^	86.06 ± 10.52 ^c^
	4.0	100.00 ± 3.66 ^c^	71.11 ± 8.63 ^a^	100.00 ± 1.02 ^c^	100.00 ± 11.21 ^cd^
T (°C)	10	88.28 ± 2.31 ^a^	100.00 ± 4.77 ^a^	91.86 ± 1.12 ^b^	72.24 ± 5.20 ^a^
	15	87.95 ± 2.41 ^a^	83.29 ± 4.82 ^b^	100.00 ± 1.14 ^a^	81.39 ± 5.24 ^ab^
	20	99.07 ± 2.29 ^b^	78.95 ± 4.66 ^b^	88.77 ± 1.16 ^c^	100.00 ± 5.35 ^bc^
	25	100.00 ± 2.42 ^b^	49.86 ± 4.90 ^c^	97.94 ± 1.16 ^b^	90.69 ± 5.38 ^b^

Relative activity values (%) and their 95% confidence intervals are shown. Different letters in columns mean significantly different at *p* ≤ 0.05.

## Data Availability

Not applicable.
